# Variety Differentiation: Development of a CRISPR DETECTR Method for the Detection of Single Nucleotide Polymorphisms (SNPs) in Cacao (*Theobroma cacao*) and Almonds (*Prunus dulcis*)

**DOI:** 10.1007/s12161-023-02500-w

**Published:** 2023-06-09

**Authors:** Nils Wax, Farshad La-Rostami, Chenyang Albert, Markus Fischer

**Affiliations:** Hamburg School of Food Science, Institute of Food Chemistry, University of Hamburg, Grindelallee 117, 20146 Hamburg, Germany

**Keywords:** CRISPR-Cpf1 DETECTR, Food fraud, CRISPR-LFA, Cocoa, Almond

## Abstract

To prevent food fraud, products can be monitored by various chemical-analytical techniques. In this study, we present a CRISPR-Cpf1 DETECTR-based assay for the differentiation of plant ingredients in sweet confectionary like fine and bulk-cocoa, or bitter and sweet almonds. To enable rapid in-field analysis, the *trans*-cleavage activity of the Cpf1 enzyme was used to develop a DETECTR (DNA endonuclease-targeted CRISPR *trans* reporter) assay for simple, highly specific fluorometric detection of single nucleotide polymorphisms (SNPs). The endonuclease Cpf1 requires the protospacer adjacent motif (PAM) 5’-TTTV-3’ for activation, but the recognition sequence is freely programmable. The SNPs were selected to alter the Cpf1 specific PAM sequence. As a result, sequences that do not carry the canonical PAM sequence are not detected and thus not cut. The optimized system was used for both raw material and processed products such as cocoa masses or marzipan with a limit of detection of 3 ng template DNA. In addition, we were able to implement the system in the context of an LFA (lateral flow assay) to serve as a basis for the development of rapid test systems.

**Supplementary Information:**

The online version contains supplementary material available at 10.1007/s12161-023-02500-w.

## Introduction

Food fraud can be defined as the intentional adulteration of food in order to obtain economic advantages (Spink and Moyer 2011). Over the past 6 years, reported food fraud cases in the European Union have more than doubled (157 in 2016 vs. 349 in 2020). The majority of the cases were based on mislabeling (37%) or substitution (21%) of the food product (European Union 2021). These numbers illustrate that fraudulent activities are a problem in the European food sector, especially with regard to the authenticity of food. To ensure compliance with European food legislation, products and their product labeling are monitored and controlled by various chemical-analytical and document-based procedures. However, documentation is prone to errors and intentional falsification, which is why chemical evaluation is the method of choice. Chemical evaluation of the biological identity (e.g., species, variety) of food products is often based on the analysis of proteins or the DNA sequences (Ballin and Laursen 2019). While the analysis of proteins in food is suitable for fresh products, this is not always the case for processed foods (Nader et al. 2013). Here, DNA-based methods are more suitable because the DNA sequence is stable during processing and even small amounts of DNA (1 ng–1 μg), can be sufficient for analysis (Primrose 2019). As DNA contains the unique genetic information of the entire organism, the genetic profile can be used to unambiguously determine the biological identity of the food product (Teske et al. 2020). In general, the more evolutionarily distant the samples, the greater sequence differences in their DNA can be expected (Barajas et al. 2019). For example, animal and plant foods are easily distinguished from each other at the taxonomic level of order, genus, and species by DNA sequence comparisons (Ho and Chan 2017). Moreover, also closely related organisms like subspecies (e.g. plant varieties, animal breeds) still show sufficient sequence differences for differentiation. These single nucleotide polymorphisms (SNPs) can then in principle be used for differentiation (De Wever et al. 2019). SNP detection is characterized by their low error rate compared to other molecular markers (De Wever et al. 2019). Furthermore, detection methods for SNPs have the advantage that standard laboratory equipment like a real-time cycler may be sufficient in many cases (Teske et al. 2020). In a previous study, Scharf et al. (2020) used the CRISPR-Cas9 (clustered regularly interspaced short palindromic repeats and CRISPR-associated protein 9) technology to distinguish SNPs of bulk- and fine-cocoa (*Theobroma cocoa*). SNPs that resulted in alteration of the motif: 5’-NGG-3’, which is the canonical PAM (protospacer adjacent motif) region of Cas9, were selected (Scharf et al. 2020). However, using Cas9 from *Streptococcus pyogenes* only one suitable SNP was available for differentiation by CRISPR-Cas9 technology (Herrmann et al. 2014). To obtain more available targets, we previously used a CRISPR-Cpf1 (from *Prevotella* and *Francisella*) (Jinek et al. 2012) based approach, which increased available detection sites on the AT-rich plastid genome in *T. cocoa* (La-Rostami et al. 2022).

The Cpf1 protein, also known as Cas12a, recognizes thymidine-rich PAM regions (5’-TTTV-3’), making this endonuclease preferable for use in AT-rich sequences (e.g. plastid genome) (Fu et al. 2019; Zaidi et al. 2017). We were able to develop two independent test systems to distinguish two cocoa varieties (fine cocoa cultivar Arriba, bulk cocoa variety CCN-51) (La-Rostami et al. 2022). With this system, admixtures of 5% CCN-51 (*P* < 0.01) and 10% Arriba (*P* < 0.05) could be detected, and successfully applied to processed cocoa products as well. Subsequent to DNA extraction and amplification by PCR, using the CRISPR-Cpf1 system led to a time reduction of 83 minutes (90 min (Cas9 system) to 7 min (Cpf1 system)). However, following the enzyme dependent digestion, templates still had to be qualitatively assessed by agarose gel electrophoresis (AGE) (Southern 1975), which took another 45 minutes. Quantitative estimations were performed by capillary gel electrophoresis (CGE) (Williams and Soper 1995) which took approximately 1 h. Comparable SNP genotyping assays for cocoa on real-time cycler systems do not need additional detection steps and are performed in about 30 minutes (De Wever et al. 2019). Hence, to further reduce the required time and infrastructure and enable in-field diagnosis, a method independent of labor-intensive detection steps and expensive devices should be used.

The CRISPR-Cpf1 system is one of the class 2/type V CRISPR-systems, (O’Connell et al. 2014) which have already been shown to be a powerful genome editing tool (Feng et al. 2013; Vestergaard et al. 2014; Zetsche et al. 2015). The Cas12a endonuclease cleaves the targeted DNA on the non-targeted and targeted strand via a single active site in the RuvC domain (Stella et al. 2017; Swarts et al. 2017). Once Cas12a enzyme binding occurs, the endonuclease exhibits *trans*-cleavage activity toward ssDNA (single stranded DNA) (Li et al. 2018). Based on this observation the so-called CRISPR-DETECTR (DNA endonuclease-targeted CRISPR trans reporter) system was developed. Used first as a sensitive DNA detection method of human papillomavirus (HPV) in patient samples and later for coronavirus (Chen et al. 2018; Huang et al. 2020), this system has been widely used in the field. Similar to the Cas13a based SHERLOCK (specific high-sensitivity enzymatic reporter unlocking) system, which has collateral activity toward RNA, *trans*-cleavage activity provides a disruption of the interaction between the fluorophore and quencher of a reporter, resulting in a fluorescent signal (Kellner et al. 2019). Furthermore, a method based on the CRISPR-Cpf1 system for on-site detection of wildtype and mutated SARS-CoV-2 by lateral flow stripes and of pathogenic microorganisms by a gold-nanoparticles-based colorimetric assay has been described, allowing naked-eye detection (Ooi et al. 2021; Yuan et al. 2020).

**Fig. 1:**
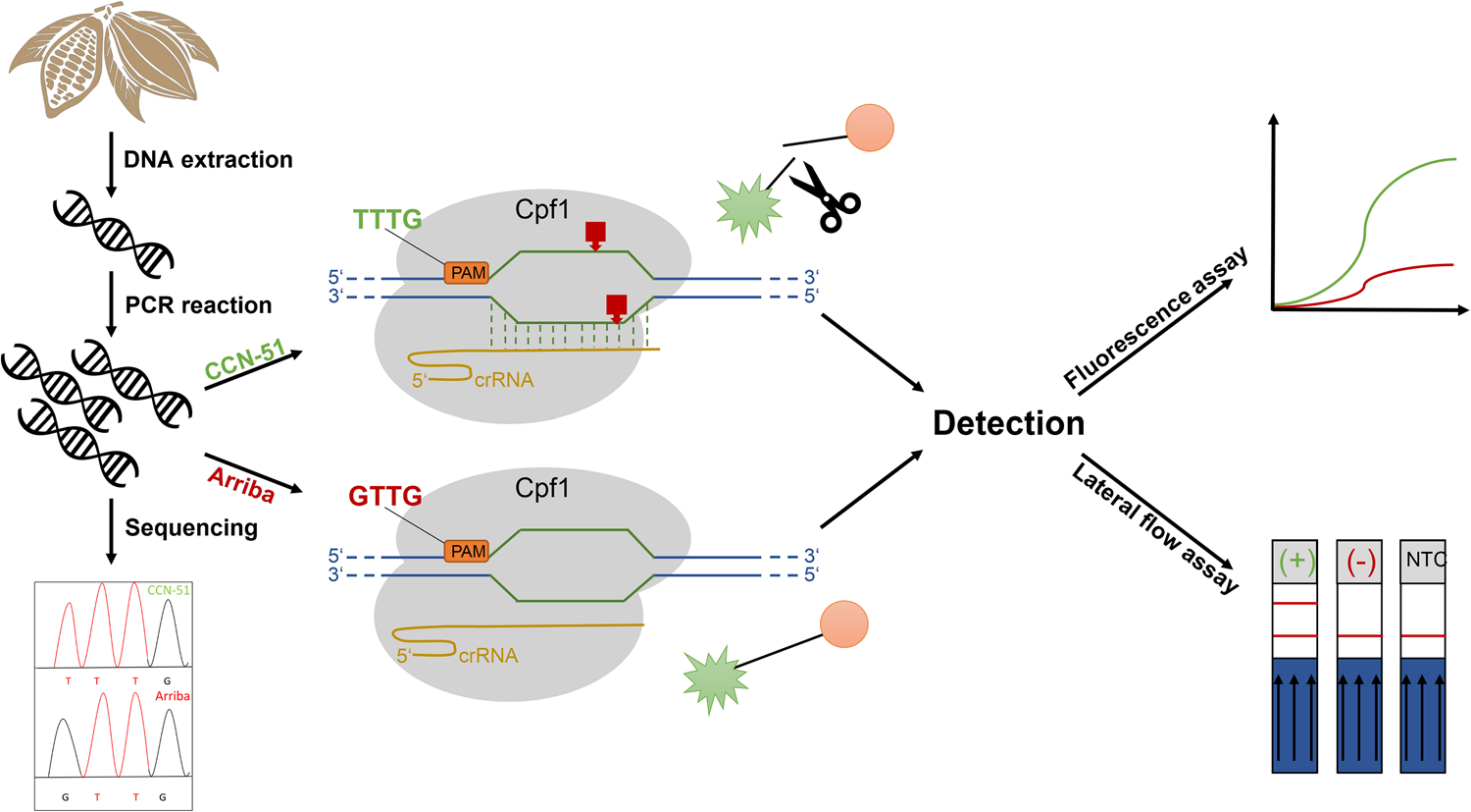
Schematic representation of the CRISPR-Cpf1 DETECTR assay using the nt 75587 locus as an example for the differentiation of CCN-51 and Arriba. First, DNA is extracted from CCN-51 and Arriba beans and amplified via PCR. The PCR products were checked for the presence of SNPs in the nt 75587 locus via Sanger sequencing. Subsequently, the CRISPR-Cpf1 DETECTR assay is performed with the amplicons. If the target sequence carries a canonical PAM (5’-TTTG-3’, CCN-51), activation of the trans-cleavage activity of the Cpf1 endonuclease occurs and the reporter is cut. If the target sequence carries a non-canonical PAM (5’-GTTG-3’, Arriba), no activation of trans-cleavage activity occurs, and the reporter remains intact. Reporters are selected according to the desired detection method. For the fluorescence assay, the reporter consists of a fluorophore (FAM) bound to a quencher; as soon as a cut is induced, a detectable fluorescence signal is generated. For the detection with bare eye via a lateral flow assay, the reporter consists of FAM bound to biotin. (+): positive control (-): negative control, NTC: no template control

In this study, we present a CRISPR-Cpf1 DETECTR-based assay to detect SNPs for authentication (Fig. 1) in bulk and fine cocoa and bitter and sweet almonds for marzipan. Due to its independence from time-consuming visualization procedures such as AGE or CGE, the test described here ensures a considerable time saving. Moreover, since no further equipment is required for detection, the application could enable a more cost-effective approach to food authenticity monitoring in routine analysis than established methods.

## Materials and Methods

### Sample Material

CCN-51 and Arriba cocoa beans from Ecuador were used. The genotype was verified by Sanger sequencing (Figure S1) with published primer for 75587 and 129451 (La-Rostami et al. 2022). To test for applicability on processed food cocoa mass of CCN-51 and Arriba was used. Sweet almonds of the cultivar Nonpareil and bitter almonds (origin: Morocco) were used for almond kernel testing. The genotype was verified by Sanger sequencing (Figure S2) with primer for *P. dulcis* locus 118388 (see Table 1). To test for applicability on processed food, marzipan rawpaste made with sweet almonds was used, as well as self-made rawpaste made with bitter almonds. Bitter almond rawpaste was prepared in our own laboratory by mixing 52% bitter almond homogenate with 37% powdered sugar, and 11% water. The obtained paste was heated for 2 h at 105 °C in a drying oven. All sample material was kindly provided by Lübecker Marzipan-Fabrik v. Minden & Bruhns GmbH & Co. KG (Stockelsdorf, Germany).

**Table 1:** Primer sequences for cocoa Loci 75587 and 129451 (La-Rostami et al. 2022) and almond locus 118388

Species	Locus	Primer	Sequence (5‘–3‘)
*T. cacao*	75587	75kFW	GTAGTTCTTCCGCTTCCAGG
		75kRV	GCCGCTTCAATGGGATCTTTT
	129451	129kFW	AAACAAAATCTTCTCTCCCCC
		short129kRV	ATGGCAGTCGTTTCAGTATGGT
*P. dulcis*	118388	118FW	ATGGTTCTCAGAAATGCCAG
		118RV	TGCATTACCGGGCATGAG

### DNA Isolation

Cocoa: DNA was isolated using the peqGOLD Plant DNA Mini Kit (Peqlab Biotechnologie GmbH, Darmstadt, Germany) according to the manufacturer’s protocol for fresh and frozen samples.

Almonds: 0.1 g of homogenized sample material of either sweet or bitter almond kernel or marzipan rawpaste were used for DNA-isolation. A CTAB-based DNA-extraction with subsequent silica purification steps was performed as described by Brüning et al. (2011). DNA was eluted with 50 μL of ultrapure water (Milli-Q, Merck KGaA, Darmstadt, Germany).

### Template Generation by PCR

All primers used to generate the templates are shown in Table 1. PCR was performed as follows: 1 U Taq DNA Polymerase and 1X Taq DNA Polymerase Buffer (both from Biozym Scientific GmbH, Hessisch Oldendorf, Germany), 1.0 μM forward and reverse Primer (IDT Integrated DNA Technologies Inc., Leuven, Belgium), 0.8 mM dNTPs (Carl Roth GmbH + Co. KG, Karlsruhe, Germany), 2 ng of template DNA, and RNase-free water (QIAGEN GmbH, Hilden, Germany) to a total volume of 40 μL. PCR was performed in a T3 Thermoblock (Biometra GmbH, Göttingen, Germany) with the following temperature program: initial denaturation step at 94 °C for 300 s, 35 cycles of (40 s at 94 °C, 40 s at 63 °C, 40 s at 72 °C), and a final elongation for 300 s at 72 °C. The purified PCR templates (see “General methods and material” section) were stored at 4 C until further use.

### Agarose Gel Electrophoresis (AGE)

For agarose gel electrophoresis (AGE) prestained agarose gels with a concentration of 1.5% agarose solved in TAE buffer (40 mM Tris acetate, 2 mM EDTA, pH 8.0) were used. Samples were mixed with 2 μL Loading Dye (40 mM Tris acetate, 2 mM EDTA, 50% glycerol, 0.005% xylenecyanol, pH 8.0). Separation was carried out at 120 V for 50 min. Results were documented under UV-light in the Dark Hood DH-40/50 and analyzed with associated ArgusX1 software (biostep GmbH, Burkhardtsdorf, Germany).

### General Methods and Material

All PCR templates were purified using the Monarch PCR and DNA cleanup kit from New England BioLabs Inc. (Frankfurt am Main, Germany) according to the manufacturer’s protocol. DNA quantity was measured fluorometrically using the QuantiFluor dsDNA System (Quantus Fluorometer, Promega GmbH, Mannheim, Germany). DNA quality was determined based on the 260 nm/280 nm ratio using the Nanodrop One^C^ (Thermo Fisher Scientific Inc., Waltham, USA). All PCR templates were stored at 4 °C. Sanger sequencing was performed by an external sequencing service (Eurofins Genomics GmbH, Ebersberg, Germany).

### Target Selection

Target selection was performed according to La-Rostami et al. (2022). A potential target must meet the following criteria: (i) SNP is located in the PAM-region, and (ii) SNP leads either to a non-canonical PAM sequence, where the RNP complex binds to the non-mutated sequence and not to the sequence with the SNP, or SNP leads to a canonical PAM sequence, where the RNP complex binds to the mutated sequence and not to the non-mutated sequence.

Cocoa: The chloroplast genome sequences (cp Genome) of Arriba and CCN-51 of Herrmann et al. (2014), were used to find appropriate SNPs. All sequences used as templates in this study can be found in NCBI under the accession numbers OM436774-OM436777 (Locus nt 75587; 5’-TT[T/G]G-3’) and OM416212-OM416215 (Locus nt 129451; 5’-[T/G]TTG-3’).

Almonds: Chloroplast genome sequences of sweet and bitter almond of the BioProject PRJNA789770 were used to find appropriate SNPs. All sequences used as templates in this study can be found in NCBI under the accession numbers OP169145-OP169149 (Locus nt 118388; 5’-TTT[T/G]-3’).

### CRISPR-DETECTR System

All crRNAs were purchased from IDT (Integrated DNA Technologies, Inc., Leuven, Belgium) and were chemically modified to reduce degradation by RNases. The crRNAs (Table 2) consist of a target-specific protospacer domain (21 nt for cocoa and 22 nt for almond crRNA) and a constant loop domain (20 nt). All in vitro CRISPR-Cpf1 digestion experiments were performed with the Alt-R A.s. Cas12a (Cpf1) V3 Nuclease from *Acidaminococcus* sp. (Integrated DNA Technologies, Inc., Leuven, Belgium). First, 2.25 μl of crRNA (10 μM) was mixed with 120 ng of Cpf1 endonuclease, forming the RNP complex. The reaction mixture is composed of the formed RNP complex, 2 μL of reaction buffer (10 mM NaCl, 10 mM Tris-HCl, 10 mM MgCl_2_, 100 μg/mL BSA, pH 7.9 (New England BioLabs Inc., Frankfurt am Main, Germany)), and 30 ng of PCR template. A DNA reporter (TTATTATT) (Broughton et al. 2020) was added to the reaction mixture, which was modified at the 5' end with the fluorophore FAM (6-carboxylfluorescein) and at the 3' end with a quencher (BHQ-1) (IDT Integrated DNA Technologies Inc., Leuven, Belgium) for all fluorescence assays. The fluorescence assay was performed on the CFX96 Touch Real-Time PCR System (Bio Rad Laboratories Inc., Hercules, USA) at 25 C. Every 30 s fluorescence intensity was measured automatically to monitor *trans*-cleavage activity over time. The reaction mixture was pipetted into a 96-well plate (cat. No. 83009-668, VWR International GmbH, Darmstadt, Germany), which was sealed with an adhesive film (cat. No. 60941–078, VWR International GmbH, Darmstadt, Germany). Additionally, optimized assay conditions were repeated without a real-time cycler detection system at room temperature on a SpectraMax M5 Microplate Reader (Molecular Devices LLC., San Jose, USA). The reaction was stopped after 20 min by addition of 1 μL Proteinase K (EO0491, Thermo Fisher Scientific Inc., Waltham, USA). Fluorescence intensity was measured after 0 min, 20 min, 27 min, 30 min, and 60 min. All measurements were performed in triplicate and the corresponding mean value was calculated. The fluorescence of the sample was normalized by dividing the measured fluorescence by the detected fluorescence of the no template control (NTC). Additionally, a positive to negative sample ratio was determined (P/N ratio).

**Table 2:** Sequence for corresponding crRNA for cocoa Loci 75587 and 129451 (La-Rostami et al. 2022) and almond locus 118388

Species	Locus	crRNA	Sequence (5‘–3‘)	Length [nt]
*T. cacao*	75587	75kcrRNA	/AlTR1/rUrArArUrUrUrCrUrArCrUrCrUrU rGrUrArGrArUrUrUrUrUrCrArUrArUrArC rCrCrGrArCrArCrGrGrU/AlTR2/ (La-Rostami et al. 2022)	41
	129451	129kcrRNA	/AlTR1/rUrArArUrUrUrCrUrArCrUrCrUrU rGrUrArGrArUrArArArUrGrCrCrCrUrUrU rCrUrCrUrCrArUrArCrU/AlTR2/ (La-Rostami et al. 2022)	41
*P. dulcis*	118388	118kcrRNA	/AltR1/rUrA rArUrU rUrCrU rArCrU rCrUrU rGrUrA rGrArU rGrGrG rCrArU rUrArA rArArA rGrCrU rUrGrU rArUrC rC/AltR2/	42

### LFA

For lateral flow assay (LFA), the Milenia HybridDetect lateral flow stripes from Milenia Biotec GmbH (Gießen, Germany) were used. To detect *trans*-cleavage success, the reporter oligo was labeled with FAM and biotin (/56-FAM/TTATTATT/3Bio/, IDT Integrated DNA Technologies Inc., Leuven, Belgium) (Broughton et al. 2020). We used the optimized reaction mix obtained from the prior conducted fluorescence measurements. Subsequently, 100 μL of HybriDetect 1 assay buffer was added to the reaction mix. The lateral flow stripe was then added to the reaction tube and incubated for approximately 2 min. Visualization of the assay result was done by bare eye.

## Results and Discussion

### DETECTR Assay for Detection of Bulk- and Fine-Cocoa

In a previous study, we successfully developed two independent assays to distinguish between bulk- and fine cocoa using the CRISPR-Cpf1 system. Based on the work of Scharf et al. (2020), specific SNPs were used which led to a mutation of the PAM sequence. Therefore a 5’-TT[T/G]G-3’ SNP at nt 75587 (OM436777.1), resulting in the digestion of CCN-51 cocoa, and a 5’-[T/G]TTG-3’ SNP at nt 129451 (OM416213.1), resulting in the digestion of Arriba cocoa, were selected. Detection of the CRISPR-Cpf1 assays result was subsequently performed qualitatively by AGE or quantitatively by CGE. However, as these detection methods require a well-equipped laboratory environment and take at least one hour of processing time, they are not suitable for routine analysis. To improve the applicability of the CRISPR-Cpf1 assay for routine analysis and to see if the assay can be implemented for in-field analysis, we took advantage of the *trans*-cleavage activity of Cpf1. The advantage of a DETECTR based approach is the in-time detection during digestion of the specific template. To apply the DETECTR system a modified single stranded oligonucleotide was used to monitor the detection event of binding of the RNP complex binding to the target DNA (Wu et al. 2021). A FAM fluorophore at the 5’-end was combined with either a quencher at the 3’-end (BHQ-1) for fluorescence detection or a biotin label at the 3’-end for detection on lateral flow stripes (see Fig. 1). This eliminates the need for time consuming steps such as AGE and CGE.

For the analysis of the cocoa samples, DNA was amplified using endpoint-PCR. The usual combination of the DETECTR system with isothermal amplification (LAMP) (Ooi et al. 2021) was not possible since the selected loci were not suitable for the design of LAMP primers. Therefore, to determine whether DNA amplification was required, experiments were performed using DNA isolates. The results showed that amplification was mandatory for cocoa samples (data not shown). This could be due to the polyphenol-containing matrix of the cocoa samples (Sies et al. 2005). The bulkiness of the phenolic compounds mainly affects the structure and activity of the protein by loosening it and causing enzyme inhibition (Cirkovic Velickovic and Stanic-Vucinic, 2018). The specific primers listed in Table 1 were used for PCR of the two loci. Amplification of the nt 75587 locus resulted in a 797 bp fragment and the nt 129451 locus resulted in a 436 bp fragment. All optimization steps for the CRISPR-Cpf1 DETECTR system were performed for both loci.

#### Optimization of CRISPR-DETECTR on Real-Time Cycler

To evaluate the assay, the ratio of measured positive and negative fluorescence values (P/N ratio) was calculated by dividing the fluorescence value of the positive control by the fluorescence value of the negative control. A ratio of 1 indicates equal fluorescence signals of positive and negative control, a P/N ratio higher than 1 indicates a stronger fluorescence of the positive compared to the negative control. A fluorescence signal of the positive signal at least twice as high as the negative control (P/N ratio of >2) was favorable to ensure reliable differentiation of genotypes, although other studies described ratios of 3 and higher as optimal conditions in authentication of animal derived food (Wu et al. 2021). To assess baseline ratios, reaction conditions of the cocoa specific CRISPR-Cpf1 assay used by La-Rostami et al. (2022) was tested at 35 C. The maximum P/N ratio observed in the initial assays was 1.05 after eight minutes, which does not yet reliably differentiate the genotype (data not shown). Therefore, assay optimization was performed to improve the P/N ratio. Optimization parameters were used according to Wu et al. (2021) and Fuchs et al. (2019). We tested the fluorescence response of positive and negative controls to varied molar ratios of Cpf1 to crRNA, different Cpf1 to reporter molar ratio, and different NaCl buffer concentrations. In addition, all assays were performed at room temperature to be independent of additional heating steps.

#### Molar Ratio of Cpf1 to crRNA

**Fig. 2:**
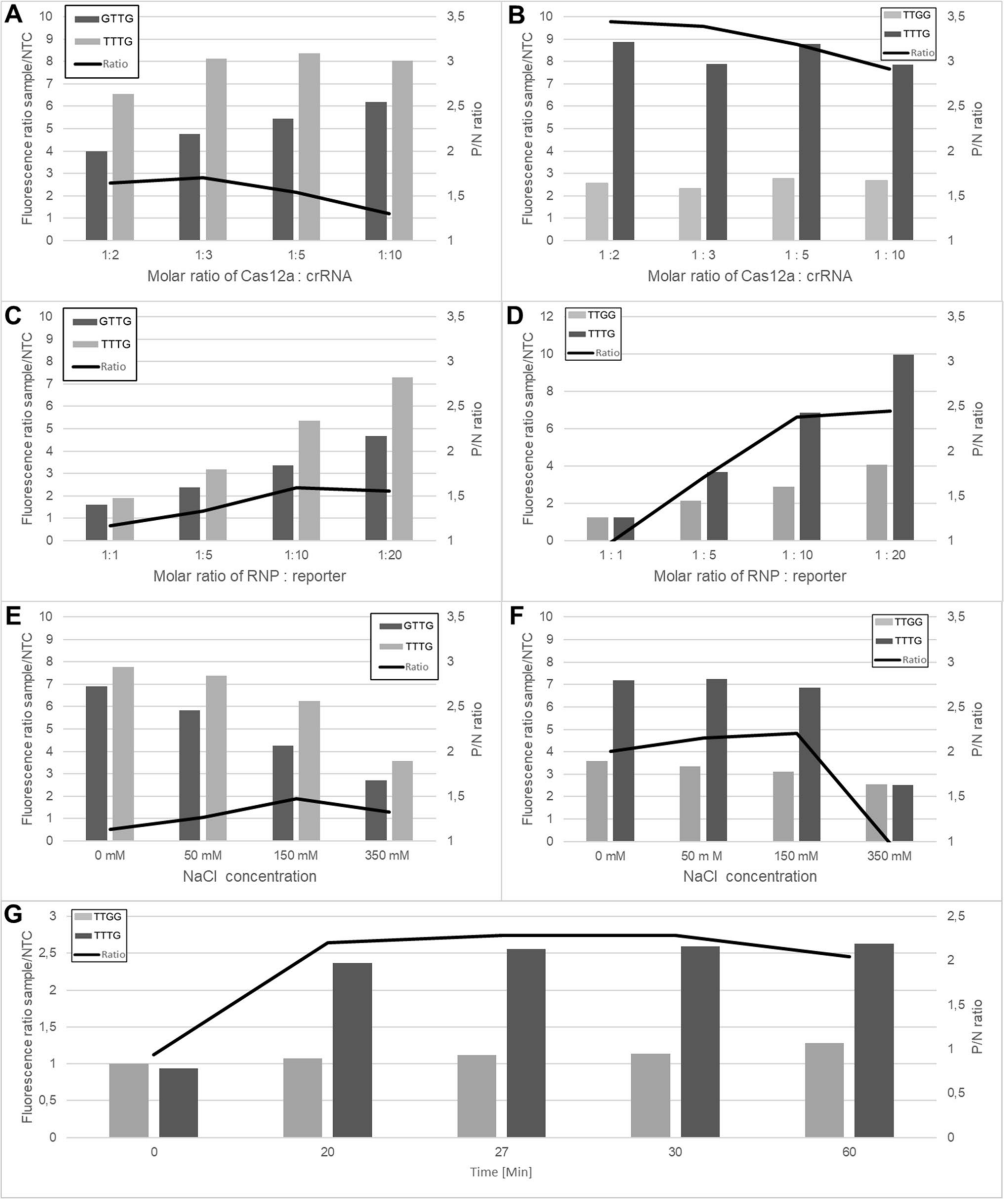
Optimization parameter of the CRISPR-DETECTR assay. CRISPR-DETECTR assay parameter were optimized for cocoa locus nt 129451 (left) and nt 75587 (right). The black line indicates the ratio of the normalized fluorescence values of measured PAM and non-PAM sequence templates. All values are given as mean value. All measurements were performed in triplicate. **A)** Molar ratio of Cas12a : crRNA for the nt 129451 locus. PAM: GTTG (dark grey bar) and TTTG (light grey bar). **B)** Molar ratio of Cas12a : crRNA for the nt 75587 locus. PAM: TTGG (light grey bar) and TTTG (dark grey bar). **C)** Molar ratio of RNP : reporter at locus nt 129451. PAM: GTTG (dark grey bar) and TTTG (light grey bar). **D)** Molar ratio of RNP : reporter for the nt 75587 locus. PAM: TTGG (light grey bar) and TTTG (dark grey bar). **E)** NaCl concentration of used buffer for the nt 129451 locus. PAM: GTTG (dark grey bar) and TTTG (light grey bar). **F)** NaCl concentration of used buffer for the nt 75587 locus. PAM: TTGG (light grey bar) and TTTG (dark grey bar). **G)** Fluorescence measurements of digested cocoa nt 75587 locus templates. The optimized CRISPR-DETECTR assay for the nt 75587 locus was used and fluorescence levels of sequences bearing the PAM or non-PAM nucleotide was measured after 0 min, 20 min, 27 min, 30 min and 60 min. Prior to the 20 min measurement 1 μl of Proteinase K was added to the samples in order to terminate the reaction. The black line indicates the ratio of the normalized fluorescence values of measured PAM and non-PAM sequence templates. PAM: TTGG (light grey bar) and TTTG (dark grey bar)

The concentration of crRNA significantly affects the cleavage of fluorescent labeled reporter oligos (Wu et al. 2021). Therefore, to assess the optimal ratio of Cpf1 to locus specific crRNA, ratios of 1:2, 1:3, 1:5, and 1:10 Cpf1 to crRNA were tested, respectively (see Fig. 2 A and B). The highest P/N ratio (1.71) for the locus nt 129451 was determined at a 1:3 Cpf1 to crRNA ratio, consistent with the results of Wu et al. (2021). However, a P/N ratio of 1:2 (3.44) was determined for locus nt 75587, with a slightly lower ration of 1:3 (3.39). A 1:2 ratio between Cpf1 and crRNA is similar to the ratio used by Broughton et al. (2020) in their DETECTR assay to detect SARS-CoV-2. Of note, lowering the temperature from 35 to 20 °C compared with the initial assays already increases the P/N ratio from 1.05 to 1.54 (129451 locus; 1:5 ration Cpf1 to crRNA) and 3.19 (75587 locus; 1:5 ratio Cpf1 to crRNA).

#### Molar Ratio of RNP and Reporter-Oligo

To find an optimal ratio of RNP and reporter oligos, different ratios were tested (1:1, 1:5, 1:10, 1:20). The results showed that 1:20 ratio gave the highest P/N ratio for both loci, corresponding to 750 nM reporter oligo concentration each (see Fig. 2 C and D). The observed ratio of 1:20 is higher than the Cpf1 protein to reporter ratio of 1:5 determined by Wu et al. (2021). However, our results are plausible, as Lv et al. (2021) calculated the Km of LbCas12a to be 585.40 nM.

#### NaCl Content

The next step in optimizing the CRISPR-Cpf1 DETECTR system was the adjustment of the NaCl concentration in the reaction buffer. Fuchs et al. (2019) showed that the Na^+^ concentration had a negative effect on the *trans*-cleavage activity of Cpf1. The observed effects of the salts regarding the *trans*-nuclease activity were due to modulation of the electrostatic potential required for nucleic acids to interact with the activated RNP in *trans*. Hereby, lower salt concentrations transient electrostatic interactions are more favorable than higher salt concentrations (Fuchs et al. 2019). Therefore, different NaCl concentrations (0 mM, 50 mM, 150 mM, and 350 mM) in the reaction buffer were tested (see Fig. 2 E and F). For the nt 129451 locus a decline in overall fluorescence was observed, which confirms the negative effect of NaCl on the *trans*-nuclease activity. However, up to a concentration of 150 mM NaCl the P/N ratio could be increased by the addition of salts. A P/N ratio of 1.26 for 50 mM and 1.47 for 150 mM was generated. The overall negative effect on *trans*-nuclease activity may favor the discrimination between small sequence difference present in the here selected PAM region, leading to slightly higher P/N ratios. For the nt 75587 locus, the negative effect of NaCl on the *trans*-nuclease activity was only evident from a NaCl concentration of 350 mM. Adding 350 mM NaCl to the reaction mix led to reduced overall fluorescence and a P/N ratio of 1. With lower salt concentrations, a P/N ratio of 2.16 for 50 mM and 2.21 for 150 mM was obtained. Although the results with a NaCl concentration of 150 mM give a slightly better P/N ratio, the present study was continued with the original NaCl concentration of 50 mM to ensure better comparability with data from the literature using the preferred NEBuffer 2.1 (Broughton et al. 2020; Wu et al. 2021).

#### Fluorometric Measurements

After optimizing the CRISPR-Cpf1 DETECTR system for both loci, only the nt 75587 locus revealed P/N ratios higher than 2, which was set as favorable to ensure reliable differentiation of genotypes. Hence, for further tests the nt 75587 locus specific assay was used with a molar ratio of Cpf1 to crRNA of 1:2, a molar ratio of RNP and reporter-oligo of 1:20, corresponding to a reporter concentration of 750 mM, and 50 mM NaCl. Measuring the fluorescence response of the positive and negative control to the above-described optimization steps on a real-time cycler enabled us to minutely monitor the time dependent increase of the fluorescence signal. At the time when the P/N ratio peaked, proteinase K was added to the reaction to stop the assay. In this way, a standardized fluorescence intensity could be ensured. Proteinase K has been successfully used in previous assays to stop digestion (La-Rostami et al. 2022). At the initial fluorescence determination (t= 0 min) the P/N ratio was approximately 1 (0.94) (see Fig. 2 G). When the *trans*-cleavage assay for the nt 75587 locus was stopped after 20 min with proteinase K, the P/N ratio of 2.20 corresponded to the P/N ratio of the 50 mM NaCl measurement (2.16, compare Fig. 2 F). Thus, the DETECTR assay described here yields reproducible P/N ratios regardless of which of the two fluorescence detecting devices were used to measure fluorescence. To verify the successful termination of *trans*-cleavage activity by proteinase K, additional fluorescence measurements were performed. After 27 min, and 30 min fluorescence increased slightly to 2.29 and 2.28, respectively. After 60 minutes, the fluorescence began to decrease to 2.05. This indicates a successful termination of the *trans*-cleavage activity of Cpf1 by proteinase K.

#### Detection Limits of the DETECTR Assay

To determine the detection limits of the *trans*-cleavage activity of the DETECTR assay for the nt 75587 locus, different concentrations of PCR templates were used to measure fluorescence after 20 min. Based on signal-to-noise evaluation, 3 ng of input DNA was sufficient to detect fluorescence signals higher than the NTC (see supplementary material Figure S3). Although the absolute fluorescence value continuously increased after 20 min, the P/N ratio did not further improve. The highest P/N ratio and absolute fluorescence values were obtained with 30 ng of input DNA. The performance of the cocoa specific DETECR assay is consistent with the performance of the published porcine-specific DETECR assay (2.7 ng/μL) in terms of detection limit (Wu et al. 2021).

### DETECTR Assay for Detection of Sweet and Bitter Almonds

In Germany chocolate often contains whole almond-kernels as an ingredient and the popular confectionary marzipan is often covered with chocolate. Therefore, it would be convenient to provide a test-system to easily monitor food authenticity of both cocoa and almonds. According to German food law, almonds are the kernel of sweet almond only and the usage of debittered bitter almonds in marzipan is not allowed (Bundesministerium für Ernährung und Landwirtschaft 2021). To see if the optimized assay is also suitable to detect almond adulterations in-field, we applied the assay to single sweet and bitter almond kernels. Chloroplast genome sequences of sweet and bitter almond of the BioProject PRJNA789770 were used to find appropriate SNPs. The sequence adjacent to a [G/T] substitution at nt 118388, resulting in a change in the canonical PAM 5’-TTT[G/T]-3’ in Moroccan bitter almonds, was used to design locus specific crRNA (see Table 2 for sequence). Digestion of total DNA with crRNA at locus nt 18388 and optimized assay parameters resulted in P/N values of 2.6 and 1.93 for 50 mM and 150 mM NaCl, respectively (see supplementary material Figure S4). Thus, 50 mM NaCl ensured the highest P/N ratio in almond isolates. Unlike the DETECTR-assay for tested cocoa loci, the assay specific for almonds did not need prior amplification by PCR when DNA was extracted using the method described by Brüning et al. (2011).

### Detection of Successful Trans-cleavage with LFA

**Fig. 3:**
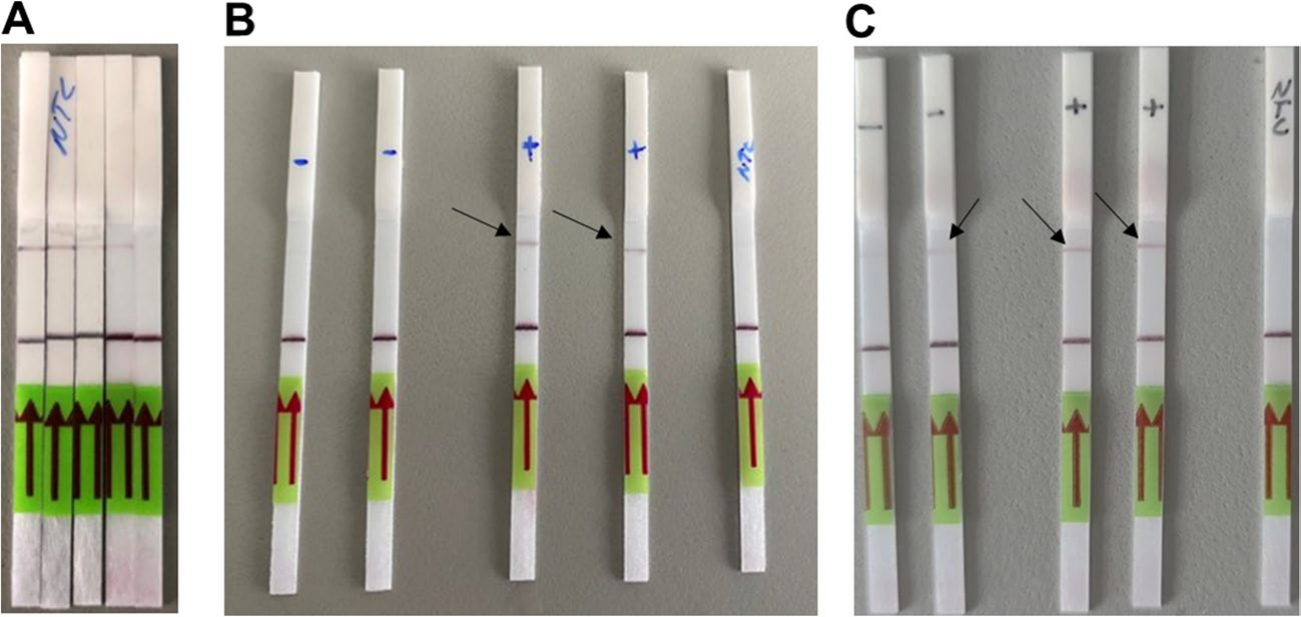
Results of the Lateral flow assay. **A)** NTC reactions with different concentration of reporter oligo; from left to right: 125 nM, 250 nM, 375 nM, 500 nM, 625 nM **B)** (+), **CCN-51**; (-), **Arriba**; NTC, no template control **C)** (-): **sweet almond**; (+): **bitter almond**; NTC, no template control. Black arrows highlight the visible test line of the LFA

To ensure the optimal concentration for the biotin labeled reporter oligo, different oligo concentrations were tested in no template control reactions (Fig. 3 A). As the amount of reporter increased, the intensity of the NTC test line decreased until no test line was visible at 625 nM reporter concentration. The reporter concentration observed here is consistent with those reported by Li et al. (2022). In the next step, detection of successful *trans*-cleavage of the optimized DETECR assay for the nt 75587 specific cocoa locus and the nt 118388 specific almond locus was tested on LFA-stripes (Fig. 3 B for cocoa and C for almond). The intensity of the positive control test line was clearly visible in the cocoa LFA, while the test line of the negative and NTC samples remained negative. In comparison, the intensity of the test line of the bitter almond sample was less intense than the test line of the positive cocoa test stripes. Additionally, a weak band was observed for the sweet almond LFA dipsticks, which was not apparent for NTC. Published results showed that slightly visible test bands of negative samples may be acceptable if the normalized intensity of the band is above that of the background (NTC) (Ooi et al. 2021). The difference between both positive bitter and negative sweet almond sample was sufficiently visible. Hence, the detection of the CRISPR-Cpf1 *trans*-cleavage with LFA dipsticks worked well for cocoa and almond samples.

### Authentication of Processed Products

**Fig. 4:**
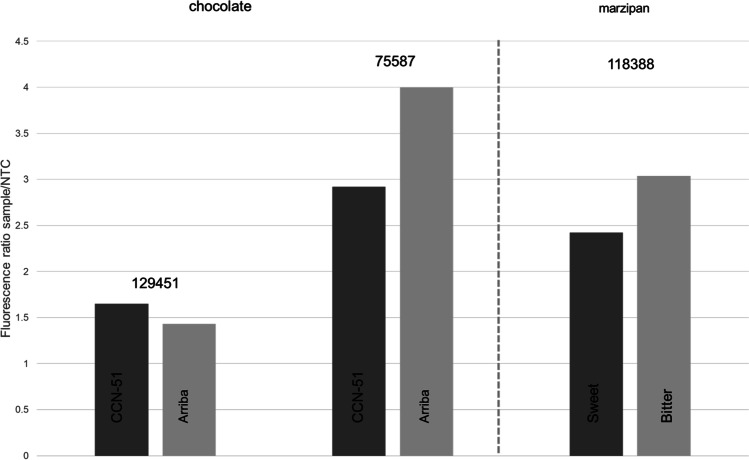
Fluorescence measurements of digested DNA of fine and bulk cocoa mass (chocolate) and digested DNA of sweet and bitter marzipan raw paste

We have shown that the CRISPR-Cpf1 DETECTR system is suitable for the differentiation of fine- and bulk cocoa as well as sweet and bitter almonds in unprocessed raw materials and hence, also works for closely related species. Since the production of chocolate as well as marzipan is a multi-step process in which the DNA under investigation is partly fragmented, the CRISPR-Cpf1 DETECTR assay was also optimized for processed products (Bernardo et al. 2005; Mano et al. 2017). Processed products were prepared for the CRISPR-Cpf1 DETECTR assay as in previous work (La-Rostami et al. 2022). The results of CRISPR-Cpf1 DETECTR assays applied to cocoa- and marzipan masses are shown in Fig. 4. Differentiation worked, but inferior results were observed compared to when the assay was applied to raw materials. For cocoa locus nt 75587 and almond locus nt 118388 the fluorescence ratio sample/NTC was higher than 2, speaking for sufficient differences in fluorescence intensity to the NTC and successful *trans*-cleavage activity. However, for cocoa masses, a P/N ratio of 1.15 was obtained for locus nt 129451, and a P/N ratio of 1.37 for locus nt 75587. Application to marzipan resulted in a P/N ratio of 1.2. Hence, CRISPR-Cpf1 *trans*-cleavage activity was not suitable to differentiate between single nucleotide variants in the PAM region when processed products were used. The observed low P/N ratio did not allow for straight forward determination of the genotype of tested cocoa cultivars (CCN-51 and Arriba) and marzipan masses. A P/N ratio of 2 or higher was favored for reliable genotyping, which was not reached for processed samples with the here described DETECTR assays. Interestingly, processed meat products could successfully be authenticated by a *cytb* targeting DETECTR assay (Wu et al. 2021). However, differentiation was performed on a higher taxonomic rank (order), so that more sequence differences were present, and differentiation did not have to rely only on single nucleotide variants. Although the canonical PAM motif of Cpf1 extends potential target sites for the AT-rich plastid genomes, finding suitable sequence variants in closely related species can be challenging (Teske et al. 2020). Furthermore, Wu et al. (2021) showed that the location of crRNA is crucial for a high signal to background ratio, indicating that the sequence of used crRNA may have affected the success of the here presented DETECTR assays.

## Conclusions

In this work, we demonstrated that the CRISPR-Cpf1 DETECTR system is suited for SNP detection in unprocessed plant material. Two assays were developed to discriminate fine- (Arriba) and bulk (CCN-51) cocoa, and one assay to differentiate sweet and bitter almonds. Two different reporters were used, depending on the detection method. For fluorometric detection, FAM and a 3’-end quencher were added to the reporter. Upon activation of the *trans*-cleavage activity of Cpf1 caused by the presence of a canonical PAM, the reporter was cut, and a fluorescence signal was detectable. In this way, P/N ratios were generated that allowed differentiation of plant cultivars. By replacing the quencher with a biotin label, detection of the system by LFA was possible. Compared to previous methods, such as detection of the CRISPR-Cpf1 assay via AGE and CGE, the DETECTR based system proved to be much more suitable for application in routine analysis by both fluorometric detection and LFAs. The detection time could be reduced from approximately 1 h to 20 min via fluorometric detection and 30 min for LFAs. In addition, the use of the LFA system does not require an elaborate laboratory infrastructure, enabling future user-friendly on-site use for commodity monitoring. To obtain a completely laboratory-independent test system, DNA extraction for cocoa and almond and target amplification for cocoa need to be optimized for in-field testing.

## Supplementary Information

ESM 1 Figure S1: Electropherogram of Sanger sequenced A) nt 129451 and B) nt 75587 locus templates. Figure S2: Electropherogram of Sanger sequenced nt 118388 locus almond sample templates. Figure S3: Limit of detection of the optimized DETECTR assay. Figure S4: Optimized DETECTR assay tested on almond samples (nt 118388 locus). Excel-Sheet: Fluorescence raw data and calculation of sample/NTC, and P/N ratio.

## Data Availability

The sequences generated during the current study can be found in the GenBank (NCBI) database under the accession numbers OM436774-OM436777 (Locus nt 75587), OM416212-OM416215 (Locus nt 129451) and OP169145-OP169149 (Locus nt 118388) (https://www.ncbi.nlm.nih.gov/genbank/). Raw fluorescence data and calculated sample/NTC and P/N ratios described in the manuscript can be accessed via the following link: 10.5281/zenodo.7410490.

## Abbreviations

AGE: agarose gel electrophoresis
Cas9: CRISPR-associated protein 9
CCN-51: Colleción Castro Naranjal 51
cp: chloroplast
Cpf1: CRISPR-associated from *Prevotella* and *Francisella*
CGE: capillary gel electrophoresis
CRISPR: clustered regularly interspaced short palindromic repeats
crRNA: CRISPR RNA
DETECTR: DNA endonuclease-targeted CRISPR *trans* reporter
HPV: human papillomavirus
LAMP: loop-mediated isothermal amplification
LFA: lateral flow assay
NTC: no template control
PAM: protospacer adjacent motif
P/N ratio: ratio of measured positive to negative fluorescence values
SHERLOCK: specific high-sensitivity enzymatic reporter unlocking
SNP: single nucleotide polymorphism
ssDNA: single stranded DNA
