# Supplementary material for: Variety Differentiation: Development of a CRISPR DETECTR Method for the Detection of Single Nucleotide Polymorphisms (SNPs) in Cacao (Theobroma cacao) and Almonds (Prunus dulcis)
Source: Food Anal Methods. 2023 Jun 9:1–11. Online ahead of print. doi: 10.1007/s12161-023-02500-w (PMC10251332; doi:10.1007/s12161-023-02500-w)
Supplement: Supplementary file 1 — Figure S1: Electropherogram of Sanger sequenced A) nt 129451 and B) nt 75587 locus templates. Figure S2: Electropherogram of Sanger sequenced nt 118388 locus almond sample templates. Figure S3: Limit of detection of the optimized DETECTR assay. Figure S4: Optimized DETECTR assay tested on almond samples (nt 118388 locus). Excel-Sheet: Fluorescence raw data and calculation of sample/NTC, and P/N ratio. [file 12161_2023_2500_MOESM1_ESM.docx]

**Supplementary Information**

**Variety differentiation: Development of a powerful CRISPR DETECTR method for the detection of single nucleotide polymorphisms (SNPs)**

**Food Analytical Methods**

Nils Wax^1,‡^, Farshad La-Rostami^1,‡^, Chenyang Albert^1^, Markus Fischer^1,^*

^1^ Hamburg School of Food Science, Institute of Food Chemistry, University of Hamburg, Grindelallee 117, 20146 Hamburg, Germany

^‡^ These authors contributed equally to this work.


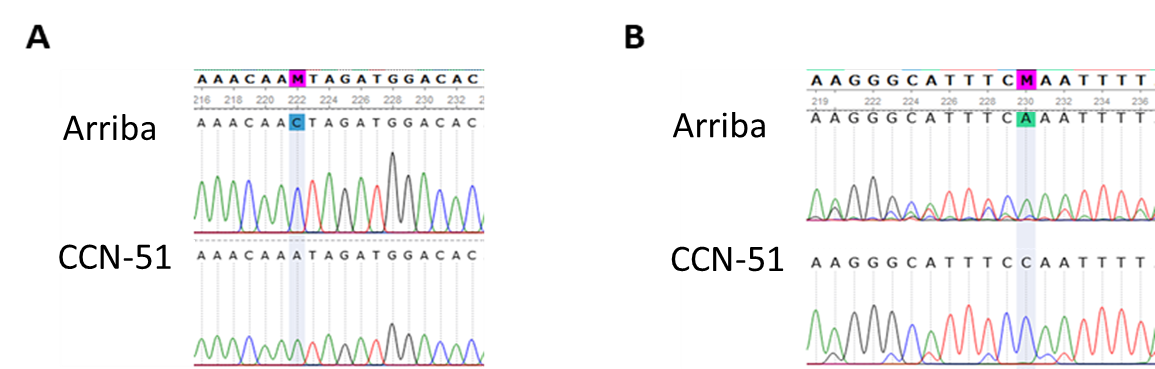


**Figure S1:** Electropherogram of Sanger sequenced A) nt 129451 and B) nt 75587 locus templates.


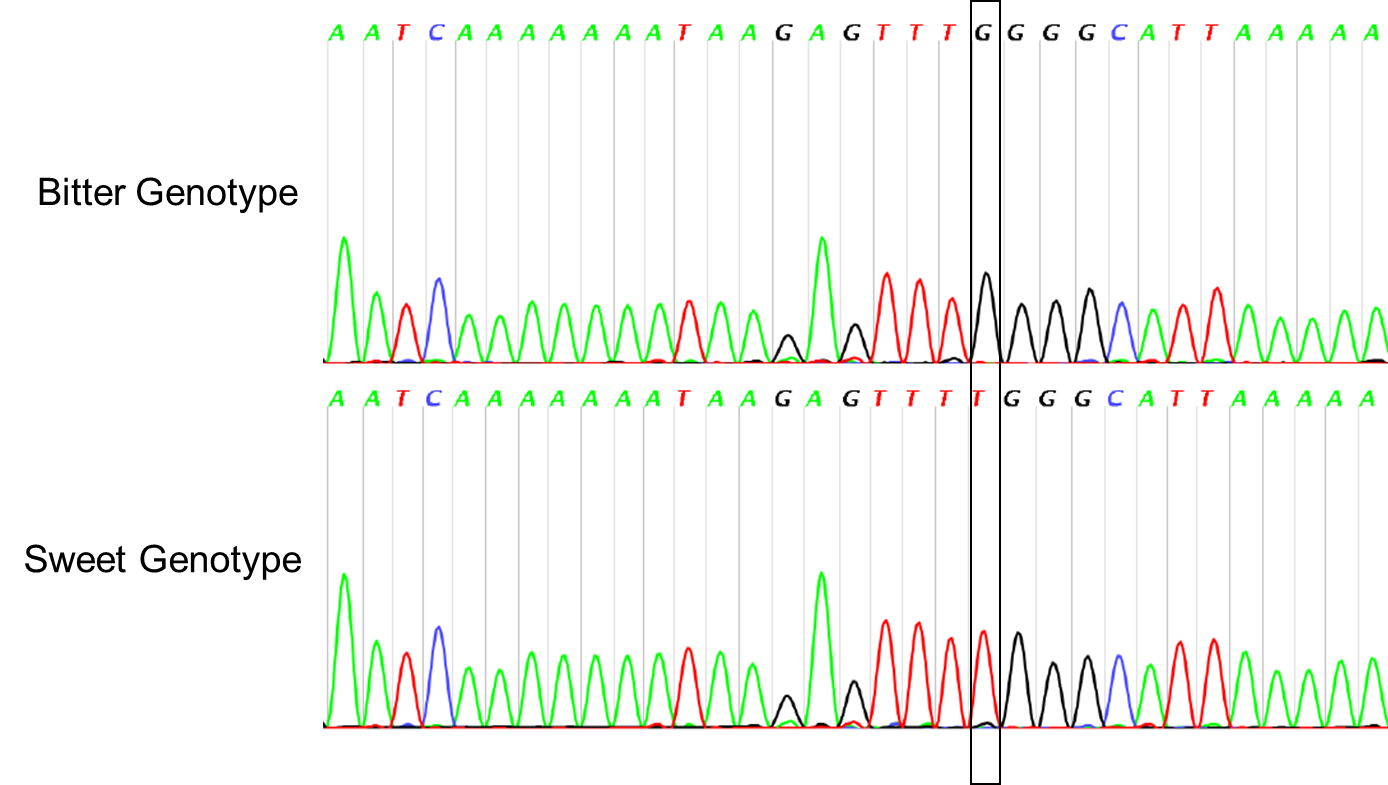


**Figure S2:** Electropherogram of Sanger sequenced nt 118388 locus almond sample templates.


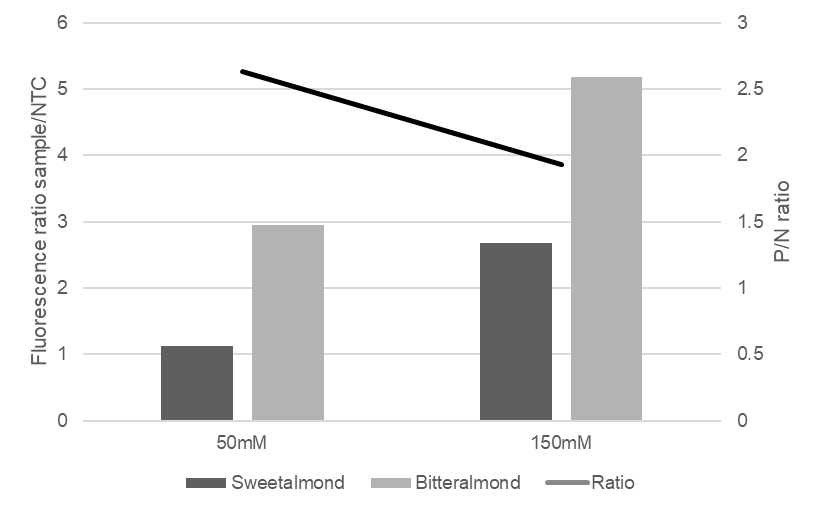


**Figure S3:** Optimized DETECTR assay tested on almond samples (nt 118388 locus). Sweet almond (PAM: TTTT, dark grey bar) and bitter almond (PAM: TTTG, light grey bar) templates were used to test the optimized DETECTR assay with concentrations of 50, and 150 mM NaCl.


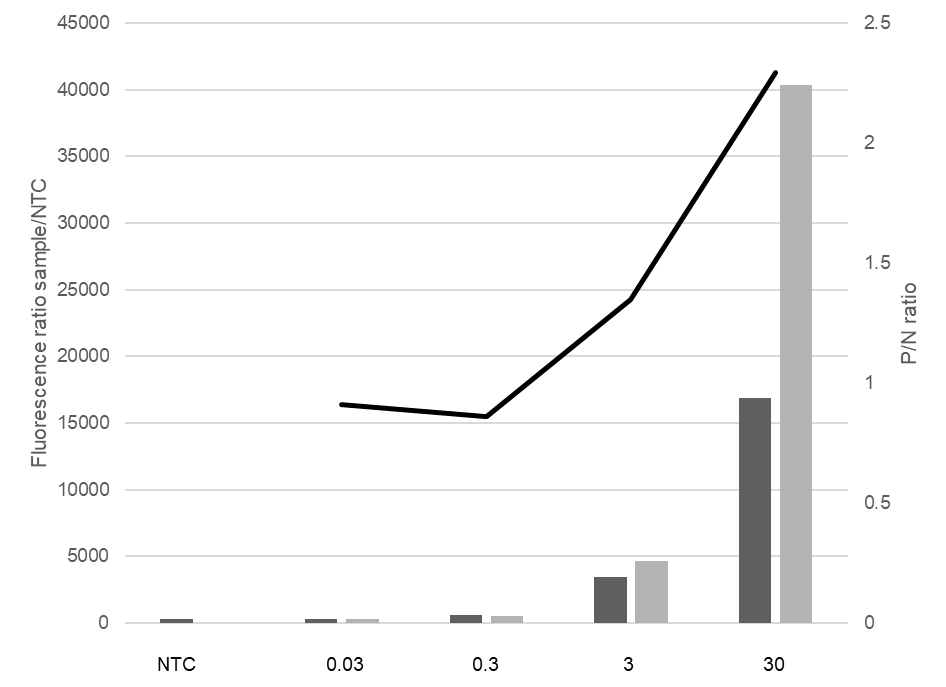


**Figure S4:** Limit of detection of the optimized DETECTR assay**.** The optimized CRISPR-DETECTR assay for the nt 75587 locus was used to determine the limit of detection. Used amount of DNA templates: 0.03, 0.3, 3, 30 ng, PAM: TTGG (dark grey bar) and TTTG (light grey bar). The black line indicates the ratio of the normalized fluorescence values of measured PAM and non-PAM sequence templates (P/N ratio). NTC: no template control.
